# CNS cell-type localization and LPS response of TLR signaling pathways

**DOI:** 10.12688/f1000research.12036.1

**Published:** 2017-07-19

**Authors:** Gizelle M. McCarthy, Courtney R. Bridges, Yuri A. Blednov, R. Adron Harris

**Affiliations:** 1 Institute for Cellular and Molecular Biology, University of Texas at Austin, Austin, TX, 78712, USA; 2 Waggoner Center for Alcohol and Addiction Research, University of Texas at Austin, Austin, TX, 78712, USA; 3 Insitute for Neuroscience, University of Texas at Austin, Austin, TX, 78712, USA

**Keywords:** Toll-like receptor, MyD88, TRIF, microglia, astrocyte, neuroimmune, lipopolysaccharide

## Abstract

**Background:** Innate immune signaling in the brain has emerged as a contributor to many central nervous system (CNS) pathologies, including mood disorders, neurodegenerative disorders, neurodevelopmental disorders, and addiction. Toll-like receptors (TLRs), a key component of the innate immune response, are particularly implicated in neuroimmune dysfunction. However, most of our understanding about TLR signaling comes from the peripheral immune response, and it is becoming clear that the CNS immune response is unique. One controversial aspect of neuroimmune signaling is which CNS cell types are involved. While microglia are the CNS cell-type derived from a myeloid lineage, studies suggest that other glial cell types and even neurons express TLRs, although this idea is controversial. Furthermore, recent work suggests a discrepancy between RNA and protein expression within the CNS.

**Methods:** To elucidate the CNS cell-type localization of TLRs and their downstream signaling molecules, we isolated microglia and astrocytes from the brain of adult mice treated with saline or the TLR4 ligand lipopolysaccharide (LPS). Glial mRNA and protein expression was compared to a cellular-admixture to determine cell-type enrichment.

**Results:** Enrichment analysis revealed that most of the TLR pathway genes are localized in microglia and changed in microglia following immune challenge. However, expression of *Tlr3 *was enriched in astrocytes, where it increased in response to LPS. Furthermore, attempts to determine protein cell-type localization revealed that many antibodies are non-specific and that antibody differences are contributing to conflicting localization results.

**Conclusions:** Together these results highlight the cell types that should be looked at when studying TLR signaling gene expression and suggest that non-antibody approaches need to be used to accurately evaluate protein expression.

## Introduction

Innate immune signaling has been well characterized in the body for decades, but the recent appreciation for its role in the brain has raised several questions. In particular, it has brought to light the similarities and differences between the immune response in the periphery and the central nervous system (CNS). At the center of this discussion are microglia, the resident immune cells of the brain. However, there is evidence that microglia have unique functions unrelated to immune signaling, and that other CNS cells can also participate in the immune response.

**Figure 1.**
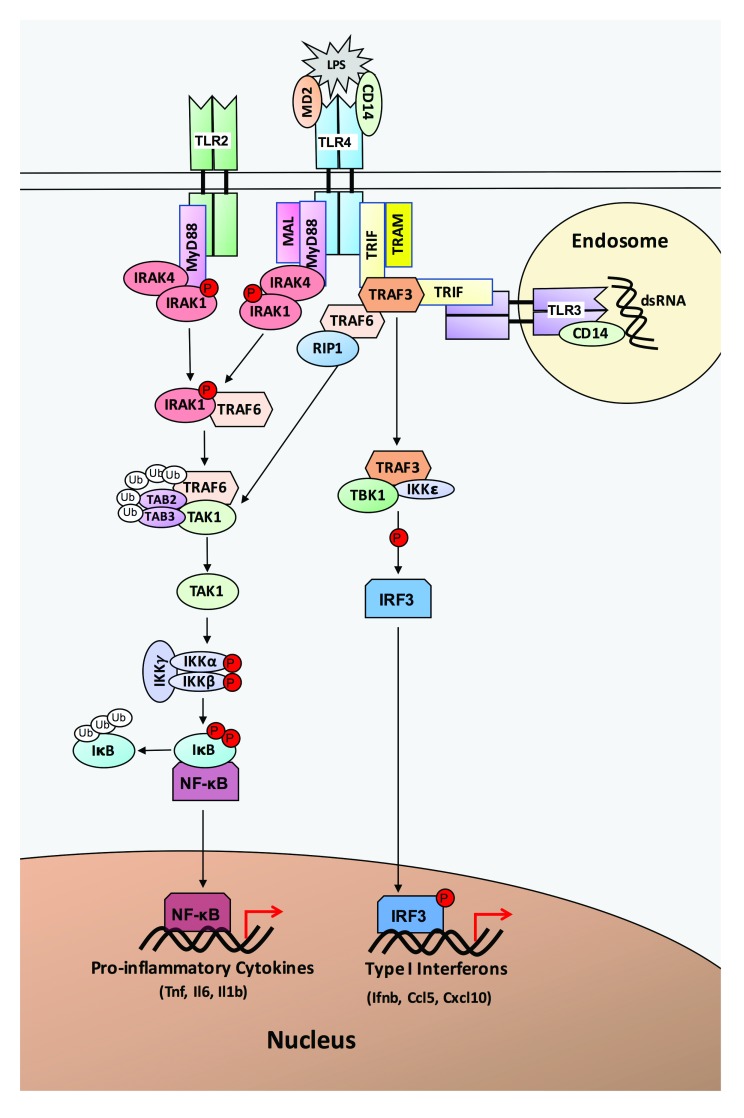
TLR-signaling pathways. Lipopolysaccharide (LPS) is recognized by TLR4 and its co-receptors MD2 and CD14. TLR4 signals through two different pathways, the MyD88-dependent pathway and the TRIF-dependent pathway. The MyD88-dependent pathway utilizes the adapter protein MyD88, which recruits IRAK4, IRAK1, and TRAF6. Phosphorylation of IRAK1 and ubiquitination of TRAF6 leads to activation of IKKs and NF-κB. Activated NF-κB translocates to the nucleus where it promotes transcription of pro-inflammatory cytokines. TLR2 also signals through the MyD88-dependent pathway. The TRIF-dependent pathway, utilized by TLR3 and TLR4, signals through the adapter protein TRIF. TRIF recruits TRAF6 and TRAF3. Signaling through TRAF6 leads to NF-κB activation, while signaling through TRAF3 utilizes IKKM *ε* to activate IRF3. Activated IRF3 translocates to the nucleus, where it leads to transcription of Type I interferons and interferon inducible genes.

A key component of innate immunity is Toll-like receptors (TLRs), a family of pattern recognition receptors that detect and respond to pathogen and danger signals. TLRs respond to a variety of bacterial and viral pathogens, including the bacterial endotoxin lipopolysaccharide (LPS), which is a ligand for TLR4 ^1^. In response to LPS, TLR4 with its co-receptor cluster of differentiation 14 (CD14) can signal through two distinct pathways, the myeloid differentiation primary response protein 88 (MyD88)-dependent pathway and the TIR-domain containing adaptor protein inducing IFNβ (TRIF)-dependent pathway ^2^ ( Figure 1). The MyD88-dependent pathway signals through Interleukin 1 receptor associated kinases 1 and 4 (IRAK1 and IRAK4) and TNF receptor associated factor 6 (TRAF6), leading to activation of inhibitors of nuclear factor κB Kinases (IKKs) ^1^. Activation of IKKs causes activation of NF-κB and the production of pro-inflammatory cytokines (e.g. TNF, IL-1β, IL-6). By contrast, the TRIF-dependent pathway utilizes the adaptor protein TRIF and signals through TRAF3, TBK1 and IKK *ε*, leading to phosphorylation and activation of interferon regulatory factor 3 (IRF3) ^3^. Activated IRF3 translocates to the nucleus where it leads to the transcription of type I interferons and interferon inducible genes (e.g. IFN-β, CCL5/RANTES, CXCL10/IP-10).

TLR signaling has been implicated in several CNS conditions, including ischemia, neurodegeneration, depression, and addiction ^4– 9^. However, the cell-type localization of TLR signaling within the CNS remains controversial and impairs our understanding and ability to develop treatments based on these signaling pathways. TLR signaling was originally characterized in peripheral immune cells; thus, it was believed that CNS expression of TLRs would be limited to microglia, the immune cells of the brain. Several studies support microglial expression of TLRs, and many reaffirm the idea that expression is completely or mostly microglial ^9– 12^. However, recent studies suggest that TLRs are also expressed and functionally important in other glial cells, such as astrocytes and oligodendrocytes ^10, 11, 13– 18^, or even non-glial CNS cells, like neurons ^19– 24^. These results are complicated by differences in methodologies across studies, including differences in: protein or mRNA; *in vivo*, primary cells or in established cell lines; species; and techniques. Interestingly, there seems to be disagreement between cell-type location of mRNA and protein expression for the same molecule, which raises many questions. For example, a brain RNA expression database shows Tlr4 as highly microglial ^25^, while the human protein atlas ( proteinatlas.org) shows it only detected in neurons ^26^. Several other TLR signaling molecules (MyD88, IRAK1, TRIF, IRF3) also show highest mRNA expression in microglia, but highest protein expression in neurons.

Although many studies have reported the localization of TLRs in the CNS, few have evaluated the expression of the downstream signaling molecules and pathway outputs that are responsible for functional changes. It also remains unclear how immune activation might change cell-type expression of TLR signaling *in vivo*, as most studies have evaluated the response to TLR agonists using cultured cells ^9, 18, 27– 29^. Recent studies suggest that established cell lines and even primary cultured glial cells don’t accurately reflect the expression profile *in vivo* ^30^.

Although this discrepancy may seem esoteric, it is a major hindrance to the study of neuroimmune signaling. In our lab alone, we have had several problematic studies because it was unclear which cell type to use for a conditional knockout or viral vector, or a gene was knocked out in microglia but couldn’t be verified on the protein level because of neuronal expression. These uncertainties not only result in wasted time and money, but also delay the discovery of important results. Given the key role of TLR signaling in CNS pathologies and the desire to manipulate and understand these pathways in the brain, it is imperative that cell-type localization of these molecules is determined and agreed upon.

Based on the disagreement in the field and preliminary results that suggested TLR-signaling mRNAs are localized in microglia while protein is localized in neurons, we sought to investigate TLR signaling localization using glial cells isolated from adult mouse brain. The goals of this study were to identify the cell-type enrichment of TLR pathway mRNAs and proteins with and without immune activation (LPS treatment), and to determine which cells exhibit expression changes following activation. There is literature supporting the idea that cell-type protein expression can change after LPS ^31^, so we hypothesized that key mRNAs will be abundant in microglia so to allow rapid translation into protein in response to immune activation. Our results revealed that mRNA was primarily microglial, although there were some differences in expression profiles, and that LPS increased mRNA expression in microglia. By contrast, our protein results were inconclusive, due to non-specific antibodies and conflicting results across antibodies for the same protein. Based on our results, we conclude that much of the disagreement in the field is due to antibody failures, and that better antibodies or alternative methods need to be developed to conclusively determine protein localization in CNS cells.

## Methods

### Ethics statement

All procedures were approved by the University of Texas at Austin Institutional Animal Care and Use Committee (animal protocol number AUP-2013-00061) and adhered to the National Instituted of Health Guidelines. The University of Texas at Austin animal facility is accredited by the Association for Assessment and Accreditation of Laboratory Animal Care. All efforts were made to ameliorate any suffering of the mice. Any mice that became too sick in response to the LPS injections were euthanized.

### Animals and LPS administration

Studies were conducted in adult (6–8 weeks old) C57Bl/6J male mice (Jackson Laboratories, Bar Harbor, ME, USA). Mice were individually housed and allowed to acclimate to upright bottles one week before the start of the experiment. The experimental rooms were maintained at an ambient temperature of 21±1°C, 40–60% humidity, and a regular light/dark schedule (7 AM–7 PM). Food and water were available *ad libitum.* The mice were randomly divided into three groups, each containing 7 LPS treated mice and 5 saline treated mice (additional mice were put in the LPS group in case of death before 24 hours) ( Figure S1). The mice were weighed, had water intake measured for two days prior to injection and then were injected with either LPS (2.0 mg/kg) or saline. Mice were weighed and water intake was measured 24-hours post-injection and the mice were sacrificed with anesthesia. Weight and water consumption data is provided in Figure S2.

### Knockout animals

Knockout (null mutant) mice for TLR2, TLR4, and MyD88 are described in 32. Briefly, the TLR2 knockout mouse was B6.129S1- *Tlr2 ^tm1Dgen^*/J (Jackson Laboratories), which has a neomycin cassette inserted in the gene, making it non-functional ^33^. The TLR4 knockout mouse was B6.B10Scn-Tlr4 ^lps-del^/JthJ (Jackson Laboratories), which has the locus containing the *Tlr4* gene deleted ^34^. The MyD88 knockout mouse was B6.129P2(SJL)- *MyD88 ^tm1.1Defr^*/J (Jackson Laboratories), and is a cross of Myd88 ^tm1Defr^ mice ( *loxP* sites flanking exon 3 of *Myd88*) with Tg(Zp3-cre)93Knw mice ^35^. RT-qPCR was used to determine the transcript expression in the knockout mice ( Figure S3). The TLR2 knockout mouse showed increased expression of *Tlr2*, which is consistent with a larger transcript being produced due to the neomycin cassette ^36^. The TLR4 knockout mouse showed no transcript expression, consistent with previous studies ^34^. The MyD88 knockout mouse showed decreased expression of *MyD88*, likely due the fact that only exon 3 is removed and the primers are not on exon 3.

### Tissue harvest and microglial isolation

Five mice per group were perfused with ice-cold saline and the brain was removed (each group was performed on a different day). The dissected tissue was pooled by treatment within group (ie. all of group 1 saline samples were combined, see Figure S1). Samples were pooled to get enough microglia for both qPCR and western blots. Approximately 1% of the minced tissue was taken as a total homogenate (TH) sample that includes all cell types. The TH was further divided into 10% for RNA and 90% for protein and centrifuged at 1000 x g for 10 minutes at 4°C. The supernatant was removed and the cells were flash frozen in liquid nitrogen. The remaining sample was used for microglial isolation, as described by Nikodemova *et al*. 2012 ^37^. Briefly, tissue suspension was enzymatically dissociated using the Neural Tissue Dissociation Kit-Papain (Miltenyi Biotec, Germany) in conjunction with Pasteur pipette manual dissociation. Dissociated tissue was passed through a 70 μM strainer (Miltenyi Biotec), centrifuged at 300 x g, and resuspended in 30% percoll (Sigma-Aldrich, St. Louis, MO, USA). The percoll-cell suspension was centrifuged at 700 x g for 15 minutes at room temperature, with the myelin fraction removed from the top fraction. Cells were washed and then incubated with CD11b MicroBeads (Miltenyi Biotec) and eluted using MS columns to collect CD11b+ cells. Cells were again divided (10% for RNA and 90% for protein) and CD11b+ cell pellets were collected by centrifugation at 300 x g for 10 minutes at 4°C and then flash frozen. The CD11b- fraction was also spun down and the pellet was resuspended in astrocyte-binding ACSA2 MicoBeads (Miltenyi Biotec). The ACSA2+ fraction was collected as the CD11b+ fraction was, and the remaining negative fraction (CD11b/ACSA2-) and the astrocyte fraction (ACSA2+) were divided (10% for RNA, 90% for protein), spun down and pellets were flash frozen.

### RNA isolation and qPCR

RNA was isolated from all four fractions (TH, CD11b+, ACSA2+, CD11b/ACSA2-) using the MagMax-96 Total RNA Isolation Kit (Thermo Fisher Scientific Inc., Rockford, IL, USA). The RNA yield was quantified on a NanoDrop 1000 spectrophotometer and assessed for quality on an Agilent 2200 TapeStation (Agilent Technologies, Santa Clara, CA, USA). RNA was reverse transcribed into cDNA using the Applied Biosystems High-Capacity cDNA Reverse Transcription Kit (Thermo Fisher Scientific Inc.). cDNA was tested for genomic DNA contamination and showed at least a 10 Cq difference between the +RT (reverse transcription) and –RT samples ^38^. Applied Biosystems TaqMan® Gene Expression Assay (Thermo Fisher Scientific Inc.) primers were used, and specific assay IDs are shown in Table S1. RT-qPCR reactions were performed using SsoAdvanced™ Universal Probes Supermix (BioRad, Hercules, CA, USA) in 10-μL reactions containing 250 pg of cDNA. All reactions were performed in technical triplicates for each biological replicate and included a negative no-template control. Samples were normalized to 18s rRNA and relative expression was determined using the CFX software version 3.1 (BioRad).

### Protein isolation and western blots

Cells or tissue were homogenized in lysis buffer (150 mM NaCl, 50 mM Tris-HCl pH 7.4, 1 mM EDTA, 1% Triton-X-100, 1% sodium deoxycholic acid, 0.1% SDS, 1X Halt Protease and Phosphatase Inhibitor Cocktail; Thermo Fisher Scientific Inc.), rocked for 30 minutes at 4°C, centrifuged for 10 minutes at 10,000 x g, aliquoted, and frozen at -80°C. HEK-293 cells were kindly provided by Dr. Mihic’s laboratory. These cells were washed with cold PBS, scraped and washed with lysis buffer, and processed as described above. Protein concentrations were determined using the DC Protein Assay (Bio-Rad). Cell lysates (20 μg for fractions, 40 ug for antibody tests) were boiled for 5 minutes, run on 4-15% Mini-Protean TGX Precast Gels (Bio-Rad), and transferred to PVDF membranes using semi-dry transfer. All fraction blots contained a control sample (mouse whole brain lysate) for normalizing across blots. Membranes were blocked with 5% dried milk in TBST (Tris-buffered saline with 0.5% Tween-20) and incubated overnight at 4°C with primary antibody ( Table S2). Membranes were washed with TBST and incubated with HRP-conjugated secondary antibodies in 5% dried milk in TBST for 1 hour at room temperature ( Table S2). Bands were visualized using Pierce ECL (Thermo Fisher Scientific Inc.) and imaged on film, using G:BOX Chemi XX6 (Syngene, Cambridge, UK). Attempts were made to identify a loading control that was equal across all cell types, but every loading control examined showed differences in expression across fractions.

### Combined fluorescent *in situ* hybridization and immunohistochemistry

The protocol was adapted from Exiqon miRCURY microRNA ISH Optimization Kit (Exiqon, Vedbaek, Denmark). Mice were transcardially perfused with 4% paraformaldehyde (PFA), and the brains were post fixed overnight in 4% PFA at 4°C and transferred to 30% sucrose overnight at 4°C. Brains were fresh frozen and coronally sectioned on a cryostat (20uM). Free-floating sections were post-fixed in 10% NBF overnight at room temperature. After three 1x PBS washes (3 minutes per wash), slices were hybridized with a double DIG-labeled custom Locked Nuclei Acid (LNA) probe (Exiqon) for 1 hour at appropriate hybridization temperature ( Table S3). Following hybridization, slices were washed in 5x SSC, 1x SSC (2 times), and 0.2x SSC (2 times) at the same temperature as hybridization for 5 minutes per wash. After a final 0.2x SSC wash at room temperature for 5 minutes, slices were blocked with blocking solution (1x PBS, 0.1% Tween-20, 2% donkey serum, and 1% BSA) at room temperature for 15 minutes. Various permeabilization steps were also tested (see source data). Slices were then incubated in anti-DIG antibody (for mRNA probe) and appropriate primary antibody for protein of choice ( Table S3) overnight at 4°C. All antibodies were diluted in antibody solution (1x PBS, 0.05% Tween-20, 1% donkey serum, and 1% BSA). After three 1x PBS-T (0.1%) washes (5 minutes per wash), appropriate secondary antibodies were applied to the slices and allowed to incubate at room temperature for 1.5 hours. After three final 1× PBS washes (10 minutes per wash), slices were mounted on charged slides and counterstained with DAPI (Fluoromount-G, Southern Biotech). Slides were visualized on a Zeiss Axiovert 200M Fluorescent Microscope and analysis was completed on Photoshop CC5 (Adobe). Probe and antibody information is found in Table S3.

### Immunohistochemistry

Brains were prepared as stated above and free-floating sections were placed into PBS. Sections were permeabilized in detergent (0.1% Triton-X-100) and blocked in 10% goat or donkey serum for 1 hour at room temperature. Antibody treatment and mounting was performed as described above. Antibody information is in Table S3.

### Statistical methods

RT-qPCR data was analyzed with a two-way analysis of variance (ANOVA) and Tukey’s multiple comparisons test. All statistical analyses were performed using Prism 7 (GraphPad Software, La Jolla, CA). All p-values are shown in Table S4.

## Results

### Fraction mRNA cell-type enrichment

**Figure 2.**
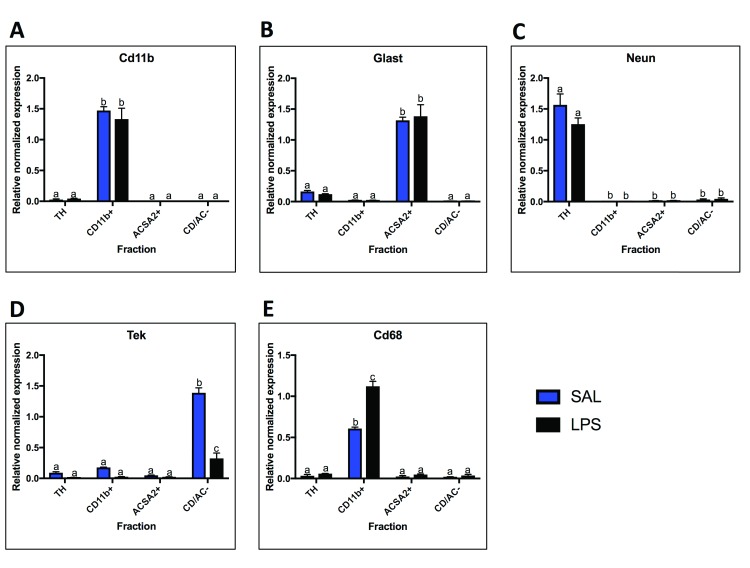
Cell-type marker mRNA expression. qPCR analysis of cell-type marker expression in the four fractions. **A.** The microglial fraction was highly enriched for the microglial marker *Cd11b*, and *Cd11b* was absent in the astrocyte and negative fraction. **B.** The astrocyte fraction was highly enriched for the astrocyte marker *Glast*, and expression of *Glast* was extremely low or absent in the microglial and negative fractions. **C.** The total homogenate (TH) had high expression of the neuronal marker *Neun*. *Neun* was absent from the microglial and astrocytes fractions and was expressed in low levels in the negative fraction. **D.** The endothelial cell marker *Tek* was highly expressed in the negative fraction and lowly expressed in the other three fractions. *Tek* expression decreased with LPS in the negative fraction. **E.** The activated microglial marker *Cd68* was highly expressed in the microglial fraction, and lowly expressed in the other fractions. *Cd68* expression increased with LPS in the microglial fraction. Two bars with the same letter are not statistically different; two bars with no letter in common are statistically different (two-way ANOVA with Tukey’s test for multiple comparisons, p<0.05). SAL, saline; LPS, liposaccharide.

Four fractions were collected from the saline and 24-hour LPS treated samples: TH (total homogenate), CD11b+ (microglial fraction), ACSA2+ (astrocyte fraction), and CD/AC- fraction (cells remaining after isolation of microglia and astrocytes, referred to as the negative fraction). RT-qPCR was performed using cell-type markers to determine the cell-type enrichment for each of these fractions ( Figure 2). *Cd11b/Itgam* was used as a marker for microglia and expression was found to be highly expressed in the CD11b+ fraction (enriched 57-fold over TH, p < 0.0001), lowly expressed in the TH, and absent in the ACSA2+ and CD/AC- fractions ( Figure 2A). *Glast/Slc1a3* was used as an astrocyte marker and was found to be lowly expressed in the TH, highly expressed in the ACSA2+ fractions under saline conditions (enriched 8-fold over TH, p < 0.0001), and absent in the CD11b+ and CD/AC- fractions ( Figure 2B). *Neun* was used as a neuronal marker and was expressed at high levels in the TH, low levels in the CD/AC- fraction (0.02-fold compared to TH, p < 0.0001), and expression was absent in the CD11b+ and ACSA2+ fractions ( Figure 2C). The reason for the lack of neuronal markers in the negative fraction is that adult neurons don’t usually survive the isolation procedure; therefore, the TH taken before isolation contains the most neurons. *Tek* was used as a marker for endothelial cells and was highly expressed in the CD/AC- fraction (15-fold over TH, p < 0.0001) and lowly expressed in the TH, CD11b+ fraction and ACSA2+ fraction ( Figure 2D). *Tek* expression decreased significantly in the CD/AC- fraction following LPS (0.2-fold, p < 0.0001). *Cd68* was used as a marker of activated microglia and was highly expressed in the CD11b+ fraction and increased following LPS treatment (1.8-fold, p < 0.0001) ( Figure 2F).

### Tlr mRNA cell-type localization and LPS response

**Figure 3.**
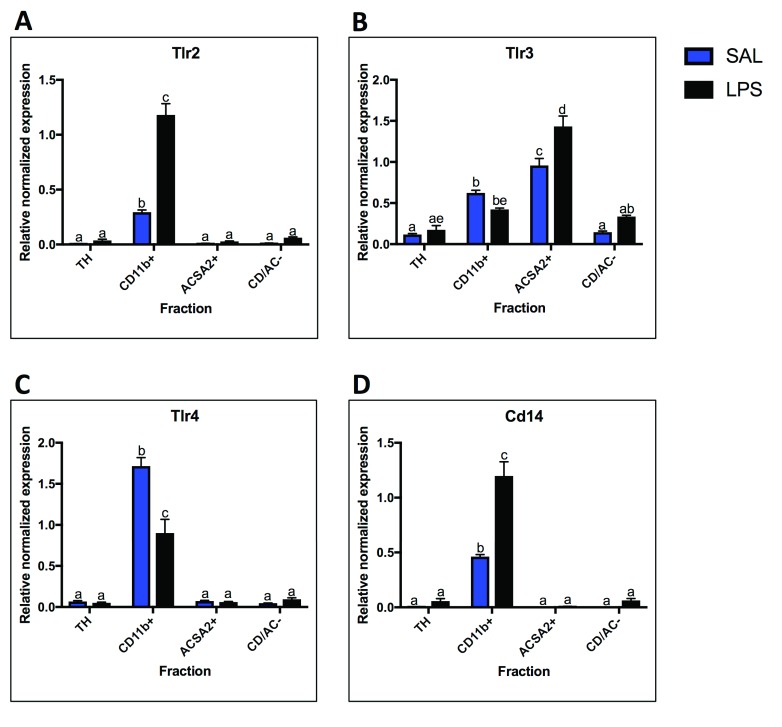
Toll-like receptor (TLR) mRNA expression. Fraction localization and LPS expression changes for TLRs and co-receptors measured by qPCR. **A.** *Tlr2* is expressed primarily in the microglial fraction and expression increases with LPS. **B.** *Tlr3* is enriched in both microglia and astrocytes compared to the total homogenate (TH), with higher expression in astrocytes. Astrocyte *Tlr3* expression increased with LPS. **C.** *Tlr4* expression is highly microglial and decreases following LPS. **D.** *Cd14* is highly enriched in microglia and increases with LPS. Two bars with the same letter are not statistically different; two bars with no letter in common are statistically different (two-way ANOVA with Tukey’s test for multiple comparisons, p<0.05). SAL, saline; LPS, liposaccharide.

qPCR was used to evaluate the expression of the most widely studied Tlrs; *Tlr2, Tlr3, Tlr4,* and the TLR4 co-receptor, *Cd14.* Under basal conditions, expression of *Tlr2*, *Tlr4* and *Cd14* was primarily localized to microglia, as evidenced by the high SAL-CD11b+ expression compared to SAL-TH expression (expression in Cd11b+ fraction over TH: *Tlr2* 41-fold, p = 0.001; *Tlr4* 25-fold, p < 0.0001; *Cd14* 75-fold, p < 0.0001) ( Figure 3). In response to LPS, *Tlr2* and *Cd14* expression increased in microglia 4-fold (p < 0.0001) and 2.6-fold (p < 0.0001), respectively ( Figures 3A and D). Alternatively, *Tlr4* expression decreased by approximately 50% in microglia following LPS (p < 0.0001) ( Figure 3C). In contrast to *Tlr2*, *Tlr4* and *Cd14*, *Tlr3* was expressed in all fractions, with highest expression in astrocytes (8-fold enrichment over TH, p < 0.0001). In response to LPS, *Tlr3* expression increased in astrocytes (1.5-fold, p = 0.0007), but not in any of the other fractions ( Figure 3B). No *Tlr* expression changes were detected in the total homogenate.

### MyD88-dependent pathway mRNA localization and LPS response

**Figure 4.**
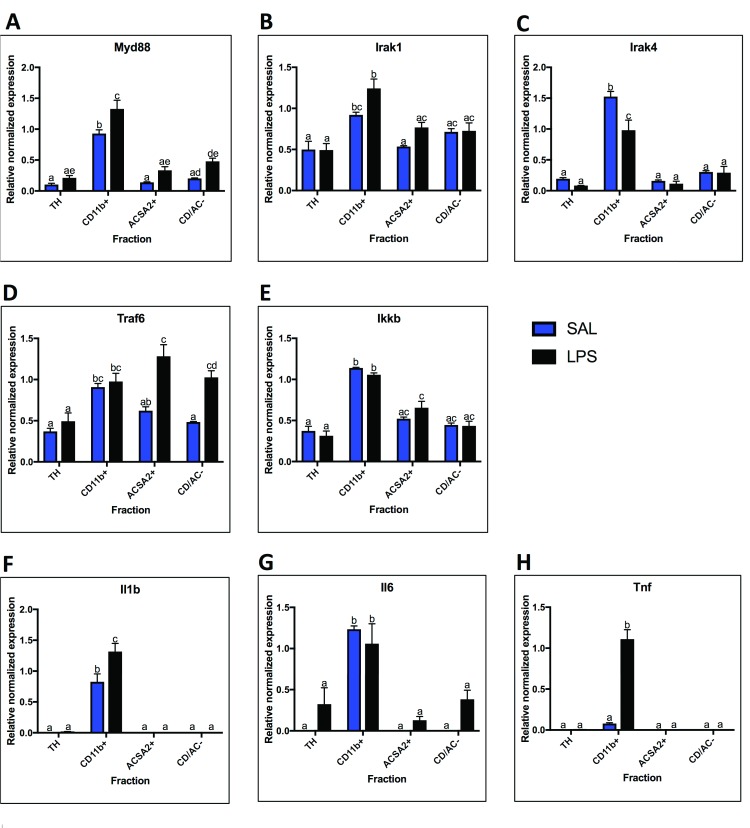
MyD88-dependent pathway mRNA expression. Fraction localization and LPS expression changes for components and outputs of the MyD88-Dependent Pathway, measured by qPCR. **A.** *MyD88* is highest enriched in the microglial fraction and increases with LPS. **B.** *Irak1* is highest enriched in the microglial fraction, but present in moderate levels (expression is 50% or more than that of microglia) in all other fractions. *Irak1* expression increases in microglia with LPS. **C.** *Irak4* expression is highly enriched in microglia under basal conditions, and decreases in microglia after LPS. **D.** With saline, *Traf6* is enriched in the microglial fraction, but present in moderate levels in all other fractions. With LPS, *Traf6* expression increases in the astrocyte fraction and the negative fraction. **E.** *Ikkb* expression was highest in microglia, but expressed in moderate levels in all other fractions. No significant expression changes were seen after LPS treatment. **F.** Expression of *Il1b* is only detected in microglia and increases with LPS. **G.** Expression of *Il6* is only detected in microglia with saline, but is detected in all other fractions after LPS. **H.** *Tnf* was only detected in the microglial fraction and increased following LPS. Two bars with the same letter are not statistically different; two bars with no letter in common are statistically different (two-way ANOVA with Tukey’s test for multiple comparisons, p<0.05). SAL, saline; LPS, liposaccharide; TH, total homogenate.

To determine localization and LPS response, mRNA expression of components of the MyD88-dependent pathway ( *Myd88*, *Irak1*, *Irak4*, *Traf6*, and *Ikkb*), as well as cytokines produced in response to MyD88-pathway activation ( *Il1b*, *Il6*, *Tnf*) were measured ( Figure 4). While all MyD88-dependent pathway genes were expressed highest in microglia under basal conditions, the expression patterns were variable. *Myd88* and *Irak4* displayed low basal expression in other fractions, while *Irak1*, *Traf6*, and *Ikkb* were expressed at greater than 50% of the expression level of microglia, suggesting expression in astrocytes and endothelial cells as well ( Figures 4A–E). In contrast, the cytokines were almost exclusively expressed in microglia ( Figures 4F–H). In response to LPS, *Myd88* expression increased in microglia (1.4-fold, p = 0.0062) while *Irak4* decreased (0.64-fold, p = 0.0023) ( Figures 4A and C). Interestingly, *Traf6* increased in astrocytes (2.1-fold, p = 0.0005) and the CD/AC- fraction (2.1-fold, p = 0.004), while *Irak1* trended towards an increase in astrocytes (p=0.02 in t-test, but not significant when corrected for multiple comparisons) ( Figures 4B and D). Both *Il1b* and *Tnf* increased in microglia following LPS administration, with *Tnf* increasing almost 14-fold ( Figures 4F and H). In contrast, *Il6* expression did not increase in microglia, but trended towards an increase in astrocytes and the CD/AC- fraction ( Figure 4G; p = 0.04 astrocytes and p = 0.03 CD/AC-, uncorrected t-test).

### TRIF-dependent pathway mRNA localization and LPS response

**Figure 5.**
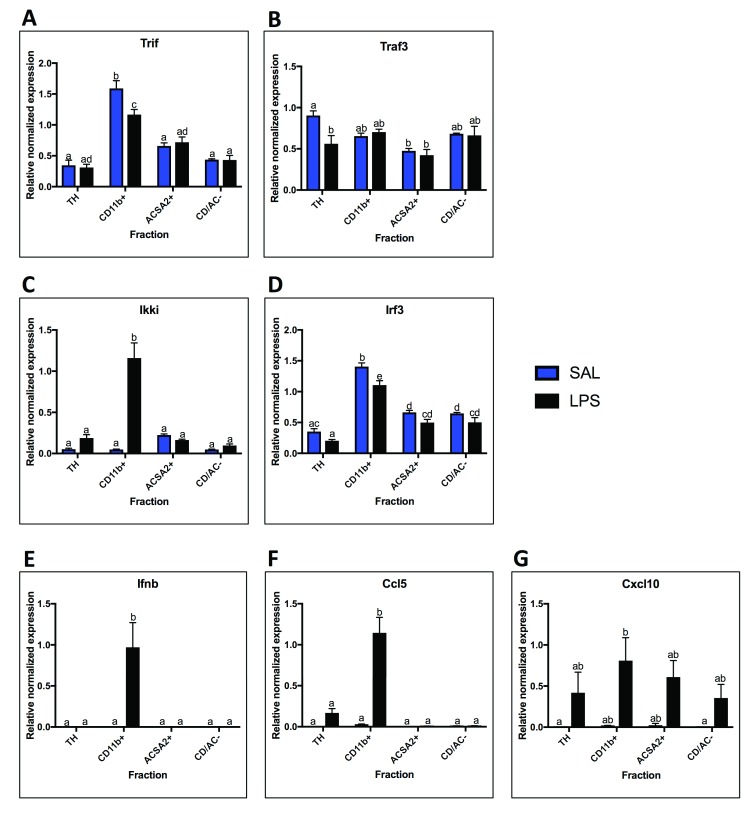
TRIF-dependent pathway mRNA expression. Fraction mRNA localization for components and outputs of the TRIF-Dependent Pathway in saline and LPS treated animals. **A.** *Trif* was highest expressed in the microglial fraction with saline and decreased with LPS. **B.** *Traf3* expression was relatively even across the fractions, with only significant difference being between the total homogenate (TH) and the astrocyte fraction. There were no significant changes with LPS. **C.** Ikki expression was not significantly enriched in any fraction with saline, but was highest in astrocytes. With LPS, expression increased in the microglial fraction. **D.** *Irf3* expression was highest in the microglial fraction, but was also significantly enriched over the TH in the astrocyte fraction and negative fraction. **E.** *Ifnb* was not detected in any fractions with saline, but was expressed in microglia with LPS. **F.** Expression of *Ccl5* was expressed in low amounts in microglia with saline, but was detected in the TH with LPS and increased in microglia. **G.** *Cxcl10* was expressed in low levels in the microglial and astrocyte fractions with saline, but was detected in all fractions with LPS, although none of the changes were significant. Two bars with the same letter are not statistically different; two bars with no letter in common are statistically different (two-way ANOVA with Tukey’s test for multiple comparisons, p<0.05). SAL, saline; LPS, liposaccharide.

Expression of TRIF-dependent pathway components ( *Trif, Traf3, Ikki*, *Irf3*) and outputs ( *Ifnb, Ccl5, Cxcl10*) were measured under basal conditions and in response to LPS to allow comparison with the MyD88-dependent pathway ( Figure 5). *Trif* and *Irf3* had similar basal expression profiles with highest expression in microglia ( *Trif* 5-fold enriched over TH, p < 0.0001; *Irf3* 4-fold enriched over TH, p < 0.0001), but *Irf3* was enriched in both the astrocyte and negative fractions (3-fold enrichment in astrocyte fraction, p = 0.01; 2-fold enrichment in negative fraction over TH, p = 0.02), while *Trif* showed only modest expression in all fractions ( Figures 5A and D). *Traf3* and *Ikki* were expressed relatively evenly across the fractions under basal conditions, although *Ikki* trended towards highest expression in astrocytes (p < 0.0001 using 1-way ANOVA for saline group) ( Figure 5B and C). Under basal conditions, *Ifnb, Ccl5, and Cxcl10* are virtually undetectable in all fractions, except for some *Ccl5* expression in microglia and some *Cxcl10* expression in microglia and astrocytes ( Figures 5E-G). In response to LPS, *Trif* and *Irf3* expression decreased in microglia ( *Trif* 0.73-fold, p = 0.02; *Irf3* 0.79-fold, p = 0.0138 *)*, while *Traf3* expression decreased in the TH (0.62-fold, p = 0.03). In contrast, *Ikki* showed 23.5-fold increase in expression following LPS (p < 0.0001). Like *Ikki*, *Ifnb* and *Ccl5* increased in microglia ( *Ifnb* only detected after LPS, *Ccl5* 37-fold increase, p < 0.0001), while *Cxcl10* trended towards an increase in microglia and astrocytes (microglia 39-fold, p = 0.047 uncorrected T-test; astrocytes 25-fold, p = .045 uncorrected T-test).

### Antibody validation in knockout tissue and HEK-293 cells

**Figure 6.**
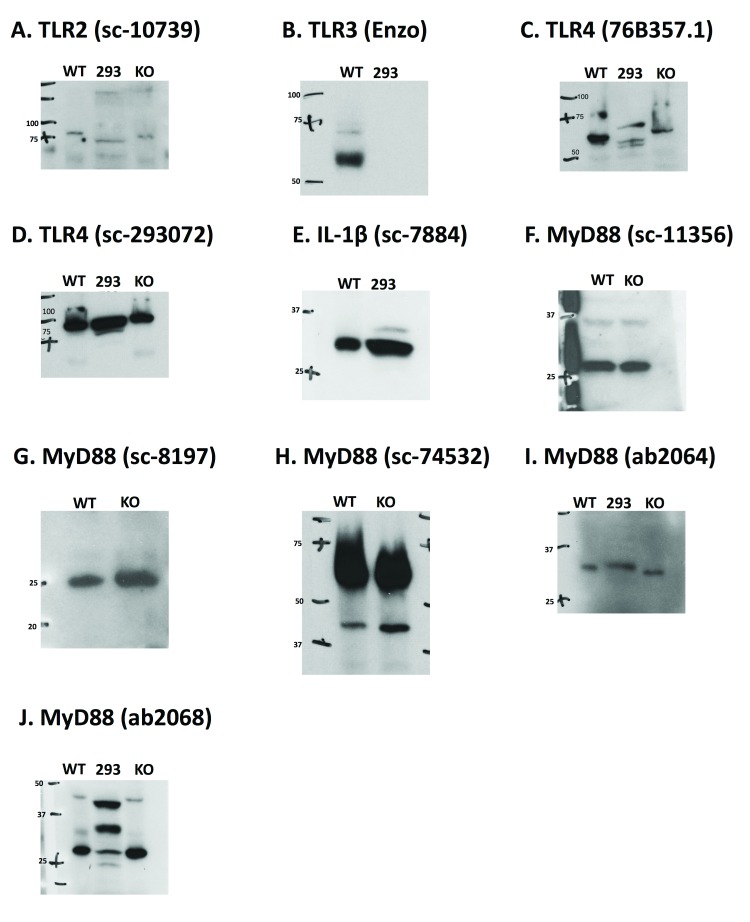
Antibody validation. Antibody tests in negative controls (knockout tissue and HEK-293 cells). Each antibody was run once with just knockout tissue if available, and once with knockout tissue and HEK 293T cell lysates. **A.** The TLR2 antibody produces a signal in HEK-293 cells and TLR knockout tissue (KO), neither of which should express TLR2. **B.** The TLR3 antibody only produced signal in the WT tissue. **C** and **D.** Both TLR4 antibodies produced signals in the HEK-293 cells and the TLR4 knockout tissue, neither of which should express TLR4. **E.** The IL-1β antibody produced a signal in the HEK-293 cells, which should not express IL-1β. **F**– **J.** All five MyD88 antibodies produced a signal in the MyD88 knockout tissue.

Knockout mice for TLR2, TLR4, and MyD88 were available in the lab and used to test the specificity of antibodies for those proteins ( Figure 6). In addition, HEK-293 cell lysates were used for validation because these cells should not express TLR2, TLR3, TLR4, or IL-1β ( www.proteinatlas.org) ^26^. Testing with the TLR2 antibody revealed expression in wild-type brain tissue, HEK-293 cells, and TLR2 knockout tissue, suggesting non-specific binding ( Figure 6A). The TLR3 antibody showed strong expression (although at a lower molecular weight than expected) in the WT brain tissue and no expression in the 293 cells ( Figure 6B). Two TLR4 antibodies were tested and both produced signals in the 293 lysates and in the TLR4 knockout tissue ( Figures 6C and D). Furthermore, the TLR4 (76B357.1) antibody appeared to run at a lower molecular weight than anticipated, though there were multiple bands that appeared at different molecular weights in each lysate ( Figure 6C). The IL-1β antibody produced a strong signal in the 293 lysates, suggesting it is also non-specific ( Figure 6E). Five MyD88 antibodies from two different companies were tested in MyD88 knockout tissue ( Figures 6F–J). All 5 antibodies produced a signal in the knockout tissue, and sc-74532 appeared at the incorrect molecular weight, indicating that none of these antibodies were specific. These tests suggested that most of the antibodies that we tested were non-specific, and made us skeptical of the ones we could not test in knockout tissue. Responses from the antibody vendors indicated that antibodies were never tested against negative controls, only against blocking peptides.

### Fraction protein localization in western blots

**Figure 7.**
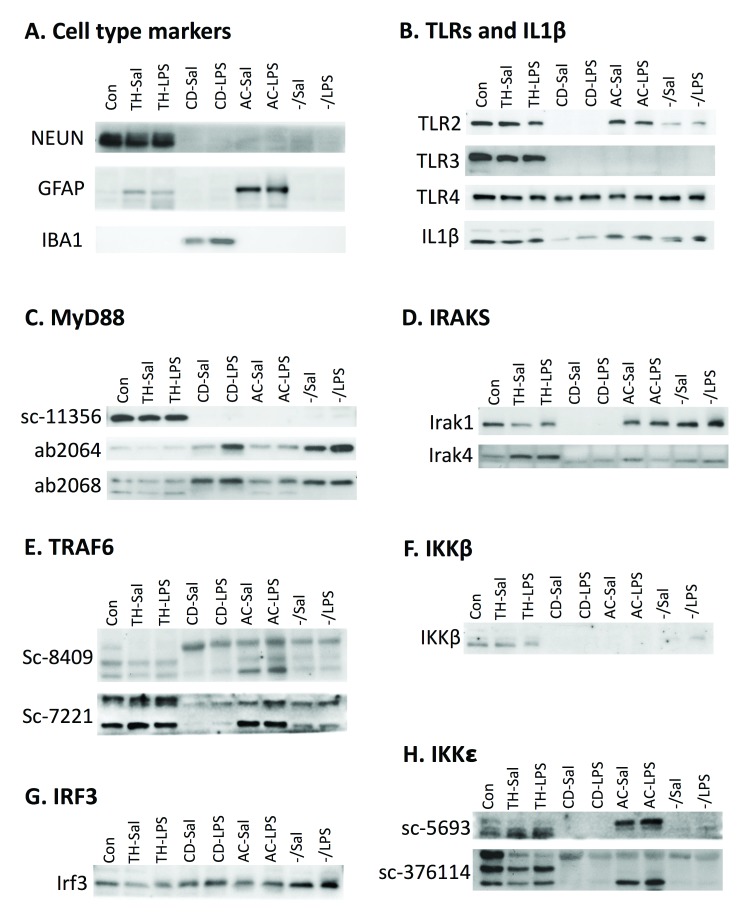
Protein expression in fractions. Fraction protein expression in representative western blot images. Number of experiments for each antibody are indicated in parenthesis. **A.** Cell-type specific antibodies verify cell-type enrichment in the fractions. NEUN, a neuronal marker, is expressed in the control sample and the total homogenate (TH) (n=3). GFAP, an astrocytic marker, is expressed in low levels in the TH and higher levels in astrocytes (n=2). IBA1, a microglial maker, is expressed in microglia (n=3). **B.** Expression for TLR2 appears to be in all fractions except microglia (n=3), while TLR3 is only detected in the TH (n=2), and TLR4 (n=2) and IL-1β (n=3) are detected in all fractions **C.** Blotting with three different MyD88 antibodies produced different results (n=3 for each). Sc-11356 suggested MyD88 is only expressed in the total homogenate, while ab2064 and ab2068 show expression in all fractions, with highest expression in microglia and the negative fraction. **D.** IRAK1 (n=2) shows expression in all fractions except microglia and IRAK4 (n=3) shows expression in all fractions, but highest expression in the TH. **E.** Two different TRAF6 antibodies produce multiple bands and different results. Based on predicted molecular weight, both antibodies show highest expression in the TH and lowest expression in microglia (n=2 for each). **F.** IKKβ showed expression in the TH and light expression in the negative fraction (n=2). **G.** IRF3 was detected in all fractions, but highest in the negative fraction. **H.** Two antibodies were used to evaluate IKK *ε*. Sc-5693 gave signal only in the TH while Sc-376114 produced signal in all cell types (n=3 for each). Sal, saline; LPS, liposaccharide; CD, CD11b+; AC, ACSA2+.

Because antibody specificity could not be verified, full replicates of western blots were not performed, and thus not quantified. However, sample western blot images for each antibody are shown in Figure 7 to demonstrate the variety of expression profiles and how different antibodies to the same protein produce different results. First, as with qPCR, cell-type marker expression was evaluated in the lysates using antibodies for NEUN (neuronal marker), GFAP (astrocyte marker), and IBA1 (microglial marker) ( Figure 7A). Differences in markers between qPCR and western blots were due to antibody availability and efficacy. Consistent with the qPCR data, NEUN was present in high amounts in the control sample and the total homogenate sample, but not in other fractions. GFAP was expressed in the control sample and the TH, but expressed highest in the astrocyte fraction, also consistent with the qPCR results. IBA1 was expressed very strongly in microglia and could be seen in the control and TH after a much longer exposure that left the microglial expression overexposed. These findings are consistent with the qPCR data which shows that expression of microglial markers is over 50x higher in the microglial fraction than the TH.

Despite the determination that many of the antibodies were non-specific, localization of TLR protein and IL-1β was investigated to see if these results mirrored some of the confusing data in the literature suggesting non-microglial localization ( Figure 7B). Even though *Tlr2* mRNA expression was predominantly microglial, TLR2 protein was detected in every fraction except microglia. TLR3, which was found to be microglial and astrocytic on the mRNA level, was found exclusively in the TH on the protein level, suggesting neuronal localization. TLR4 and IL1β were highly expressed in microglia on the mRNA level, but were expressed in all fractions on the protein level. Furthermore, IL-1β expression was lowest in microglia. These data suggest that studies detecting neuronal localization of TLRs, despite microglial mRNA, may be due to non-specific antibodies.

Because we had so many antibodies that claimed to detect MyD88, this presented an opportunity to compare localization of the same protein using different antibodies ( Figure 7C). MyD88 (sc-11356) was expressed only in the TH, suggesting neuronal expression. In contrast, ab2064 and ab2068 were expressed in all fractions, although highest in microglia and in the negative fraction. MyD88 (sc-8197) gave such strange results with vastly different molecular weight bands across the fractions, that it was not included. MyD88 (sc-74532) was not used because tests revealed that the signal was at the wrong molecular weight ( Figure 6H). These results were particularly concerning because every antibody tested in the knockout was non-specific and different antibodies produced different results.

The rest of the MyD88-pathway produced equally confusing results. Like TLR2, IRAK1 protein was expressed in every fraction except microglia. TLR4 showed highest expression in the TH, but faint expression in other fractions at a slightly lower molecular weight. For TRAF6, we had two antibodies from the same company, sc-8409 (monoclonal mouse) and sc-7221 (rabbit polyclonal). Both antibodies produced several bands ( Figure 7E), making it difficult to determine what signal was real. TRAF6 should run at 60 kD, which corresponds to the middle band on the sc-8409 blot and top and on the sc-7221 blot. Based on these bands, expression appears to be highest in the TH and the astrocyte fraction. IKKβ was primarily localized to the TH ( Figure 7F), which is consistent with the neuronal localization seen in immunohistochemistry data from our lab ^39^, but inconsistent with the qPCR data.

Protein expression evaluation was limited for the TRIF-dependent pathway (due to antibody challenges) and only expression of IRF3 and IKK *ε* was determined ( Figure 7G and H). IRF3 was expressed in all fractions, but highest expression was in the negative fraction. IKK *ε* was evaluated using two antibodies, sc-5693 (goat polyclonal) and sc-376114 (mouse monoclonal). IKK *ε* sc-5693 is a very weak antibody, but detected some protein in the control sample and total homogenate (the bands in the astrocyte fraction are suspected to be bleed through). IKK *ε* (sc-376114) is supposed to be expressed at 80 kD, which corresponds to the top band; however, the multiple bands raise concerns.

### Protein and RNA expression in tissue sections

Several of the proteins evaluated with western blot have also been investigated in brain tissue using immunohistochemistry with the same or different antibodies. Examples of these are shown in Figure S4. Immunohistochemistry reveals highly neuronal expression in tissue for MyD88 (sc-8197), IRAK1 (sc-7883), and TRAF6 (sc-7221). These results are relatively consistent across TLR-pathway antibodies that have been tested in our lab (high neuronal staining). Interestingly, attempts to look at *Irf3* mRNA expression via *in situ* hybridization also suggested neuronal localization ( Figure S5). Because we knew that *Irf3* mRNA should be in microglia, we tested a microglial marker, *Tmem119* ^40^, using the same *in situ* protocol. *Tmem119* also failed to express in microglia ( Figures S5C and D), suggesting that there may be a permeability issue when targeting glial cells in tissue, resulting in high background staining in neurons. It is worth noting that *Irf3*, which is more heterogeneous across cell types, showed a much stronger neuronal signal than *Tmem119*, which should only be in microglia. This suggested to us that the small amount of *Irf3* localized in neurons was all we could detect, while the detected neuronal *Tmem119* was just background due to increased probe concentrations.

Dataset containing the following six files: ISH localization images, ISH images, knockout animal qPCR, Sal-LPS qPCR data, Sal-LPS western blots and validation western blots Click here for additional data file. Copyright: © 2017 McCarthy GM et al. 2017 Data associated with the article are available under the terms of the Creative Commons Zero "No rights reserved" data waiver (CC0 1.0 Public domain dedication).

## Discussion

**Figure 8.**
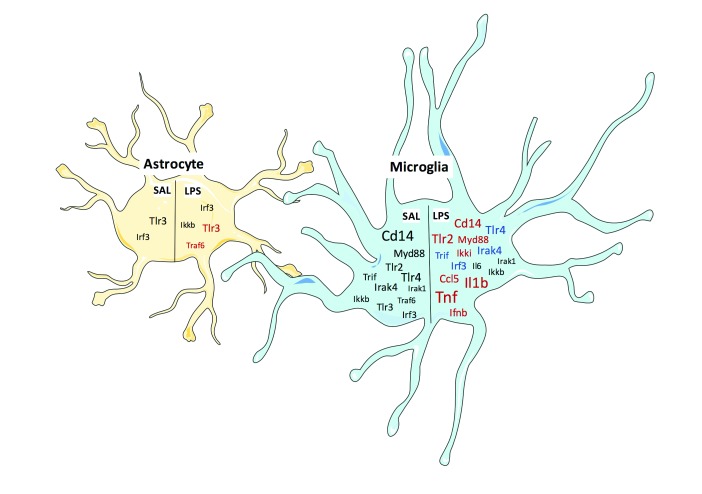
Summary of mRNA enrichment and LPS response. Microglial and astrocyte cell-type enrichment (compared to TH) is shown for TLR pathway genes in saline and LPS treated mice. The font size of each gene indicates fold-enrichment, with larger sizes meaning larger fold-enrichment. Colors on the LPS side denote whether that gene changed in that cell type with LPS treatment. Red indicates increased gene expression while blue denotes decreased gene expression. Figure created using http://servier.com/Powerpoint-image-bank.

TLR signaling is a key component of the innate immune response and it contributes to many brain disorders, including alcohol use disorders. However, the cell-type specific response to immune stimuli in the CNS remains unclear. Identification of the cell-type localization of TLR signaling and immune response within the brain is necessary to elucidate the functional implications of perturbed signaling and to design future studies with *in vivo* manipulations. To address this, we used isolated glial cells from adult mice that had been administered either saline or LPS. Using four distinct cell-fractions, we evaluated the mRNA expression of TLRs, their downstream signaling molecules, and the transcriptional outputs of their signaling ( Table 1; Figure 8). In addition, we tried to profile the protein expression of TLR signaling molecules. Unfortunately, we were not able to draw any conclusions about the protein localization, but we have identified reasons why there may be disagreement in the field.

**Table 1.** Summary of qPCR data. Summary of 24 hour LPS qPCR data. Colors indicate fraction: microglial, teal; astrocyte, yellow; negative fraction, orange. Primary localization with saline (SAL) is determined by fraction enrichment compared to the total homogenate (TH) under saline conditions, with fold-enrichment shown in the next column. Primary localization with liposaccharide (LPS) is determined by fraction enrichment compared to the TH with LPS treatment, with fold change shown in the next column. Change in each fraction with LPS is determined by comparing expression in that fraction with SAL to expression in that fraction with LPS, with direction and fold-change noted. Red indicates increased expression, while blue indicates decreased expression. Only significant differences are noted (p<0.05, two-way ANOVA with Tukey’s multiple comparisons test). CD, Cd11b+; AC, Acsa2+.

Gene	Primary localization with SAL	Fold enrichment over TH (SAL)	Primary localization with LPS	Fold change over TH (LPS)	Change in TH with LPS	Change in CD with LPS	Change in AC with LPS	Change in CD/AC- with LPS
**Toll-like Receptors and CD14**								
Tlr2	Cd11b+	41	Cd11b+	32		↑ 4.0		
Tlr3	Acsa2+	8	Acsa2+	8			↑ 1.5	
	Cd11b+	5						
Tlr4	Cd11b+	25	Cd11b+	18		↓0.53		
Cd14	Cd11b+	75	Cd11b+	21		↑ 2.6		
**MyD88-Dependent Pathway**								
Myd88	Cd11b+	9	Cd11b+	6		↑ 1.4		
Irak1	Cd11b+	2.5	Cd11b+	2.5				
Irak4	Cd11b+	8	Cd11b+	12		↓0.64		
Traf6	Cd11b+	2.5	Acsa2+	2.5			↑ 2.1	↑ 2.1
			CD/AC-	2				
Ikkb	Cd11b+	3	Cd11b+	3				
			Acsa2+	2				
Il1b	Cd11b+	not detected in TH	Cd11b+	90		↑ 1.6		
Il6	Cd11b+	not detected in TH	Cd11b+	3.3				
Tnf	none significant		Cd11b+	277		↑ 13.7		
**TRIF-Dependent Pathway**								
Trif	Cd11b+	5	Cd11b+	4		↓0.73		
Traf3	none significant				↓0.62			
Ikki	none significant		Cd11b+	6		↑ 23.5		
Irf3	Cd11b+	4	Cd11b+	7		↓.79		
	Acsa2+	3	Acsa2+	2.5				
	CD/AC-	2	CD/AC-	2.5				
Ifnb	none significant		Cd11b+	not detected in TH		only detected with LPS		
Ccl5	none significant		Cd11b+	13		↑ 36.5		
Cxcl10	none significant							

Although expression of mRNA from the TLR signaling pathway was primarily microglial as expected, there were extremely variable expression profiles within the pathways. This is consistent with gene expression data from adolescent (P17) mice in the RNA-Seq transcriptome database ^25^. While expression of *Tlr2, Tlr4, and Cd14* were highly microglial, expression of *Tlr3* was highest in astrocytes, where expression increased in response to LPS. These findings are consistent with several studies that have shown *Tlr3* to be expressed and functional in astrocytes ^15, 16, 41, 42^, as well as a study that shows *in vitro* LPS increases *Tlr3* expression in primary astrocyte cultures while decreasing *Tlr3* expression in primary microglial cultures ^18^. TLR3 signals through the TRIF-dependent pathway; however, the components of the TRIF-dependent pathway showed varied expression and LPS responses. This raises the question of how signaling molecules within one pathway could be expressed in different cell types. Although *Trif* and *Irf3* expression is highest in microglia, there is still significant expression in astrocytes, and *Ikki* expression trends towards being mostly astrocytic under basal conditions. Therefore, it is possible that signaling is occurring in both cell-types and that the mRNA expression of the receptor and its signaling molecules are not 1:1 within the cell. Furthermore, microglial *Trif* and *Irf3* expression decrease following LPS, while *Ikki* expression increases, suggesting they are independently regulated. This is supported by the involvement of *Ikki* in other LPS-responsive pathways (e.g. JAK/STAT signaling), which could have different cell-type specificity.

It is surprising that the expression of *Ifnb* and *Ccl5* is exclusively microglial. There is a trend towards increased expression of *Cxcl10* in both astrocytes and microglia after LPS, suggesting that the TRIF-dependent pathway is being activated and inducing downstream signaling in both cell types. This raises the question of how expression of *Cxcl10* is increased without *Ifnb* there to induce it. It is possible that interferon-inducible genes are produced in response to IFNβ in microglia, but changed in a different manner in astrocytes, which lack a macrophage lineage. Astrocytes produce interferon in a TLR3 and TLR4 dependent manner *in vitro* ^43, 44^, but it is possible that astrocytes respond differently *in vivo*. It is also plausible that the inflammatory response is temporally mediated within each cell-type, and that increased expression of interferons would be detected in astrocytes if evaluated earlier or later. It is noteworthy that TLR4 also signals through the TRIF-dependent pathway and is highly microglial, so perhaps TLR3 signaling is predominant in astrocytes, while TLR4-induced TRIF dependent signaling dominates in microglia, leading to the increased *Ifnb, Ccl5, and Cxcl10* seen in the CD11b+ fraction.

Components of the MyD88 pathway were highest expressed in microglia, which is consistent with expression of *Tlr2* and *Tlr4*. However, some components of this pathway ( *Irak1, Traf6, Ikkb*) were more evenly distributed across the fractions, and *Traf6* expression increased in the astrocyte fraction following LPS. The different expression profiles could be because *Traf6* can also be activated via TRIF in response to TLR3 or TLR4, and is involved in other pathways like TGF-β signaling. Additionally, *Ikkb* is involved in every pathway that signals to NF-κB, not just TLR pathways. Consistent with the notion that MyD88-dependent signaling is mostly occurring in microglia, *Il1b, Il6, and Tnf* expression were primarily microglial. However, there is a trend towards an increase in *Il6* seen in astrocytes and the CD/AC- negative fraction in response to LPS. There is evidence that *Il6* is also activated in response to LPS and TRIF-dependent signaling in cultured astrocytes ^18, 42^, so it is possible that TRIF-dependent increases in *Il6* expression occur in astrocytes *in vivo.*

It is worth noting that although several robust changes were observed in response to LPS within the cell fractions, none were observed in the total homogenate, which is the typical preparation for evaluation of gene expression. This highlights the importance of looking at discrete cell types when evaluating immune changes in the brain, particularly because expression could be decreasing in one cell type while increasing in another (as seen with *Tlr3).* A caveat to collecting cell fractions is that whole brain samples had to be pooled to get enough RNA for RT-qPCR. Because of this, any brain-region specific changes are missed and the statistical power is reduced. Furthermore, the primers used for RT-qPCR are designed to target a single exon-exon junction, so exon level expression and splice variants may be missed.

Although expression of mRNA and protein is not always 1:1, we were unable to find any examples in the literature showing all mRNA changes occurring in one cell-type and all protein changes occurring in a different cell-type. Because this is what our preliminary data suggested, we sought to test our hypothesis that many copies of mRNA were found inside microglia to ensure rapid translation in response to danger signals (although this hypothesis did not address why protein was found in neurons). After this study, we are just as unclear, if not more, about the protein localization of TLR signaling molecules. However, we do have some thoughts as to what is causing this confusion.

The western blots we performed show incredibly variable expression profiles, but the most concerning result is that several TLR signaling proteins do not appear to be expressed in microglia (TLR2, TLR3, MyD88 sc-11356, Irak1, IKKβ), even though all of them show microglial mRNA expression and most show highest expression in microglia. However, after some quality control steps, we are unable to trust any of the protein results. For the antibodies we could test, all but one showed expression in a negative control. For the antibodies we were unable to test on null mutant tissue, we erred on the side of caution and assumed they were also non-specific. Furthermore, different antibodies to the same protein gave very different expression profiles ( Figure 7C), reaffirming that the antibodies cannot be trusted. We suspect that antibody specificity is one of the major reasons for disagreement in the field. Even though other researchers have told us that TLR antibodies are notoriously non-specific, they continue to be used in publications and these results continue to be cited as accurate. Even resources like the human protein atlas use antibodies to determine cell type localization ^26^. For example, the data for MyD88 in the human protein atlas suggests that protein is highly neuronal, but RNA expression is mostly glial. Interestingly, the antibody they use is sc-11356, which we found to be non-specific ( Figure 6F). They do provide information about the antibody validation, but they are basing the validation on comparison of staining in one tissue type (colon) to the literature.

It isn’t surprising that antibodies are non-specific, given that most manufacturers only validate them in transfected cell lines or with blocking peptide. Some companies, when asked, could not even suggest a negative control and claimed it would be too difficult to test all antibodies on knockout tissue. Unless this practice changes, every lab needs to test the antibody in their hands with positive and negative controls to be confident their results are accurate. Due to the difficulty of testing several antibodies for each protein, other approaches may be better suited for looking at several proteins at once. Proteomic approaches in glial cells have revealed protein changes that more closely match what is expected ^45^. Alternatively, construction of transgenic mice with GFP-tagged expression of TLR genes may be useful to show the CNS cell-type localization.

In addition to our western blots, our immunohistochemistry and *in situ* results suggest that glial cells are less likely to be permeable to probes or antibodies. Therefore, more stringent permeabilization steps may be needed to detect intracellular molecules in glia. Although we attempted different permeabilization steps, we continued to see neuronal localization.

In conclusion, this study confirms and expands on mRNA cell-type localization of TLR signaling molecules and evaluates cell-type specific increases following LPS administration. This study was unable to reliably determine the protein localization of TLR signaling molecules, and we suggest this is due to non-specific antibodies and problems with permeabilization. We suggest that future studies evaluating cell-type expression take these results into account and that perhaps other non-antibody approaches be used to determine the protein localization of this important pathway in the CNS.

## Data availability

The data referenced by this article are under copyright with the following copyright statement: Copyright: © 2017 McCarthy GM et al.

Data associated with the article are available under the terms of the Creative Commons Zero "No rights reserved" data waiver (CC0 1.0 Public domain dedication). 

**Dataset 1. Dataset containing six files as follows:**

**IHC localization images:** This folder contains all immunohistochemistry images for MyD88, Irak1, and Traf6. The MyD88 folder contains images from 4 different MyD88 antibodies.

- Images summary: Powerpoint file containing images for neuronal staining overlayed with each antibody
- Irak1
  - Irak1 and NeuN
    ■ TIF images for Irak1, NeuN, and merged
  - MyD88
    ■ Table summarizing staining in human brain overlayed with NeuN (JPG file)
    ■ Powerpoint file summarizing MyD88 staining in primary neuronal cultures
    ■ MyD88 Abcam 2064 antibody: TIF files for MyD88, Neun, and merge
    ■ MyD88 F-19 antibody
      • MyD88 and GFAP: TIF or JPG files showing staining for MyD88, GFAP (astrocyte marker), and merge
      • Myd88 and Iba1: TIF, PSD, and JPG files showing staining for MyD88, Iba1 (microglial marker) and merge
      • MyD88 and NeuN: TIF and JPG files showing staining for MyD88, NeuN (neuronal marker), and merge
    ■ MyD88 S.Cruz Full length (HFL-296): TIF files for MyD88, NeuN and merge

**ISH images:** This folder contains *in situ* images and information

- Confocal images: Contains confocal images as TIF files showing IRF3, IBA1 and merge
- Summary of fluorescent microscope images: Contains TIF images and word documents with representative images for *in situs* (probe, protein, and merged)
- ISH testing summary: excel file containing all information about different *in situ* tests

**Knockout animal qPCR:** This folder contains Biorad CFX data files and genes study as well as GraphPad file of data and statistical analysis

**Sal-LPS qPCR data:** This folder contains all Biorad CFX data files as well as an excel file showing data from the gene study (CFX analysis for multiple plates). It also contains a GraphPad file of all data and statistical tests.

**Sal-LPS western blots:** This folder contains all raw western blot images used in Figure 7 (as TIF files).

**Validation western blots:** This folder contains all raw western blot images used in Figure 6 (as TIF files).

doi, 10.5256/f1000research.12036.d168396 ^46^

## Supplementary material

**Figure S1: Schematic of study methods.** The mice were divided into three subgroups, each containing 5 mice per treatment. Mice were injected with either saline or 2 mg/kg LPS and sacrificed 24-hours later. The whole brain was removed and tissue was pooled within each group by treatment yielding 3 biological replicates per treatment. 1% of minced tissue was taken as total homogenate and the remaining tissue was used to isolate microglia (Cd11b+) and astrocytes (Acsa2+). The remaining cells (Cd11b/Acsa2) were also collected. 10% of each sample was used for RNA isolation and RT-qPCR and 90% was used for protein isolation and western blots.

**Figure S2: Weights and water consumption after LPS.** Data verifying the effect of LPS treatment. **A.** All 3 LPS groups showed decreased weight following injection, data points are averages of 5 mice. **B.** All 3 LPS groups decreased water consumption following LPS injection, data points are averages of 5 mice.

**Figure S3: Knockout mouse qPCR.** RT-qPCR on knockout mouse brain compared to wild type (C57/Bl6J). **A.** TLR4 knockout tissue showed no *Tlr4* mRNA expression. **B.** *TLR2* knockout tissue showed an increase in Tlr2 expression. **C.** MyD88 knockout tissue showed a decrease in *Myd88* expression. * p value < 0.05, 2-tailed t-test, n =10 per group.

**Figure S4: Immunohistochemistry for MyD88, IRAK1, and TRAF6.** Representative images from immunohistochemistry evaluation of MyD88 (n =10), IRAK1 (n = 4), and TRAF6 (n=5) expression in the mouse cortex revealed co-localization with the neuronal maker NEUN. Additional images are available in the source data files.

**Figure S5: *In situ* hybridization for Irf3 and microglial marker Tmem119.** *In situ* hybridization compared mRNA expression with cell-type markers. **A.** *Irf3* mRNA shows little overlap with the microglial marker IBA1 (3 biological replicates with at least 3 technical replicates each). **B.** *Irf3* mRNA shows high overlap with the neuronal marker NEUN (2 biological replicates with 2 technical replicates each). **C.** *Tmem119*, a microglial marker, shows little overlap with IBA1 (2 biological replicates with 2 technical replicates each). **D.** *Tmem119* shows overlap with the neuronal marker NEUN (1 biological replicate).

**Table S1: Taqman gene expression assays used for RT-qPCR.**

**Table S2: Antibodies used for western blots.**

**Table S3: Immunohistochemistry and *in situ* antibodies/probes. A.** Antibodies used for immunohistochemistry, results in Figure S4. **B.** Probes used for *in situ* hybridization, results in Figure S5. **C.** Antibodies used for *in situ* hybridization, results in Figure S5.

**Table S4: Results of statistical tests.** This table contains all input data and statistical results for the qPCR data presented in the manuscript. Data in this table is extracted from the GraphPad file included in the source data.
