# Supplementary figures and images for: CNS cell-type localization and LPS response of TLR signaling pathways
Source: F1000Res. 2017 Jul 19;6:1144. [Version 1] doi: 10.12688/f1000research.12036.1 (PMC5621151; doi:10.12688/f1000research.12036.1)

Figure S1

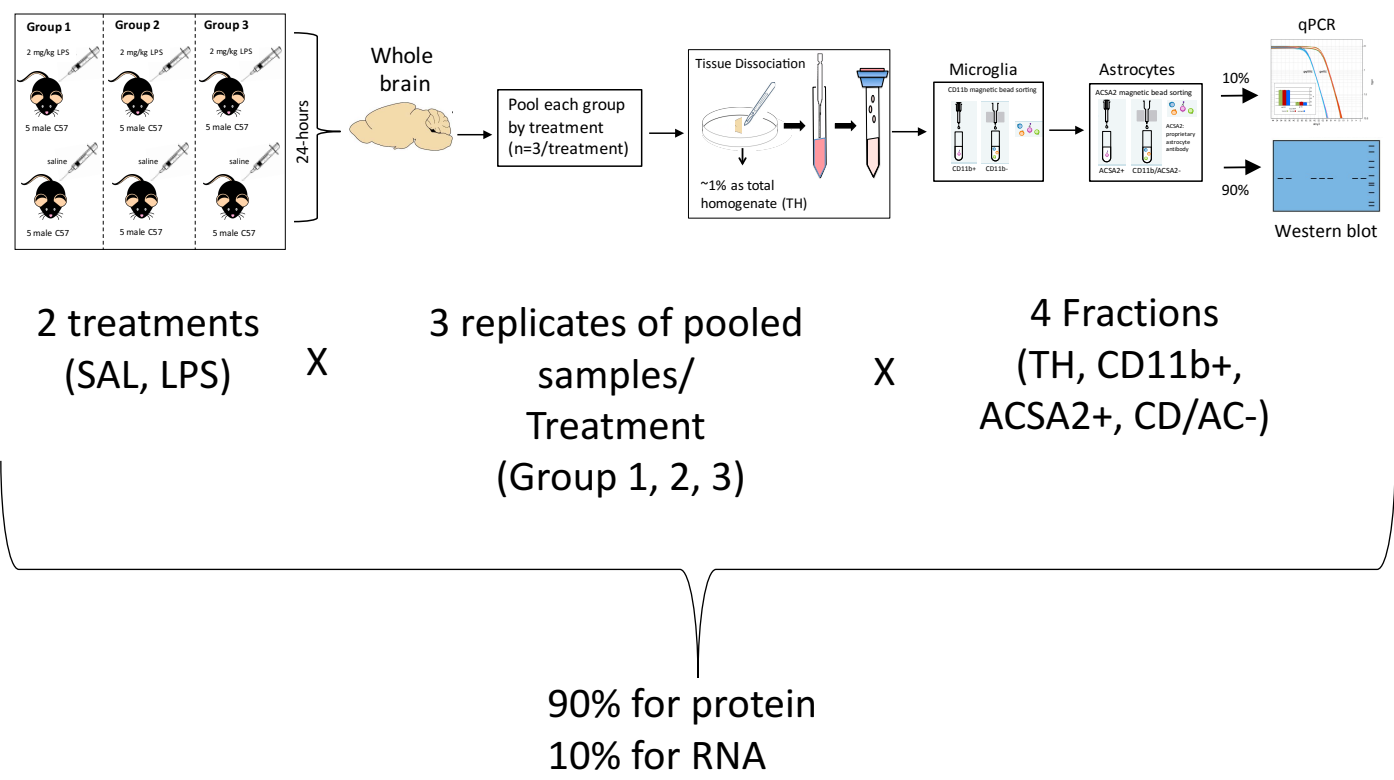

Supplement: Supplementary file 2 [file f1000research-6-13022-s0001.tgz › 6083f7ff-dd48-4aab-b402-934f9de7f703.pdf]

Figure S2

A

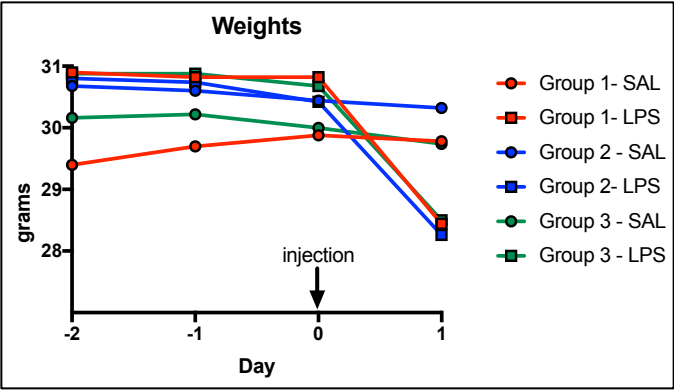

B

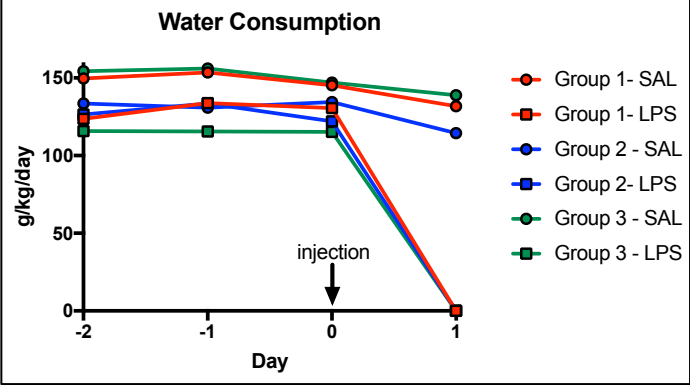

Supplement: Supplementary file 3 [file f1000research-6-13022-s0002.tgz › 2f94ec5b-aa0c-4367-b06d-efd3b6a89cd2.pdf]

Figure S3: qPCR on knockout animals

A

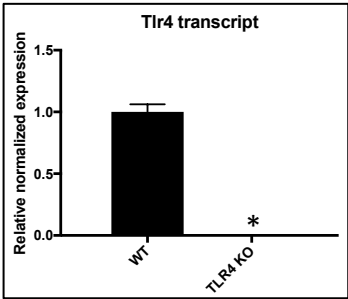

B

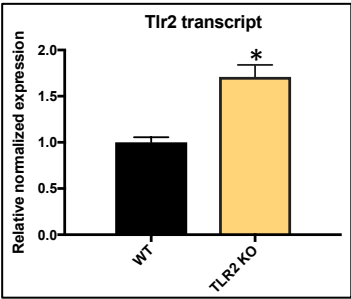

C

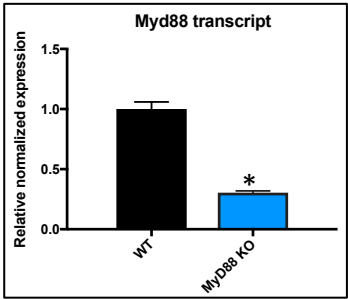

Supplement: Supplementary file 4 [file f1000research-6-13022-s0003.tgz › 30048b1d-f0ab-431e-8b3c-38979433c77b.pdf]

**Figure S4: Immunohistochemistry of some targets**

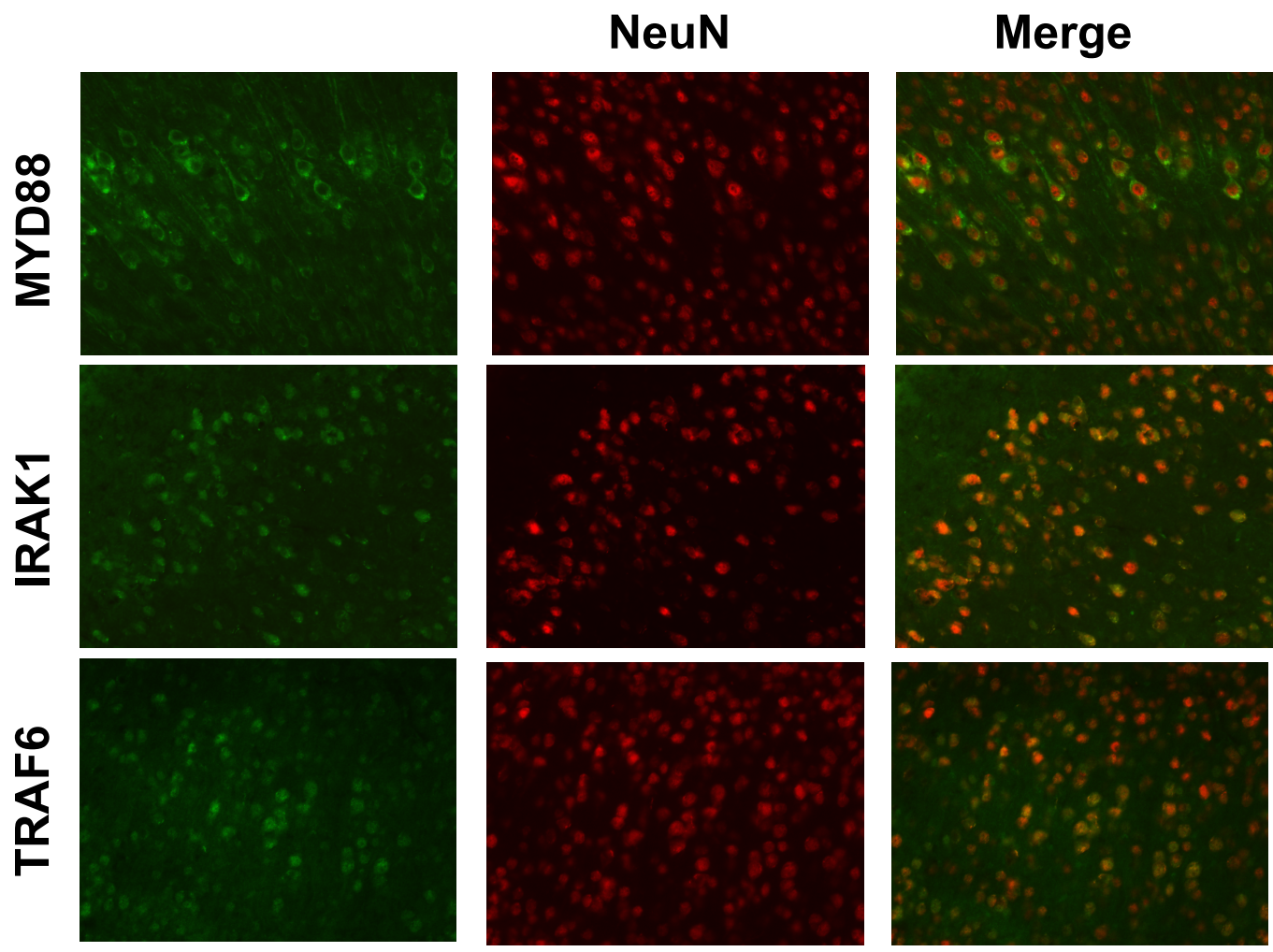

Supplement: Supplementary file 5 [file f1000research-6-13022-s0004.tgz › 45983acf-97d2-4a6b-85a9-6e055cd4cc83.pdf]

Figure S5: In situ hybridization

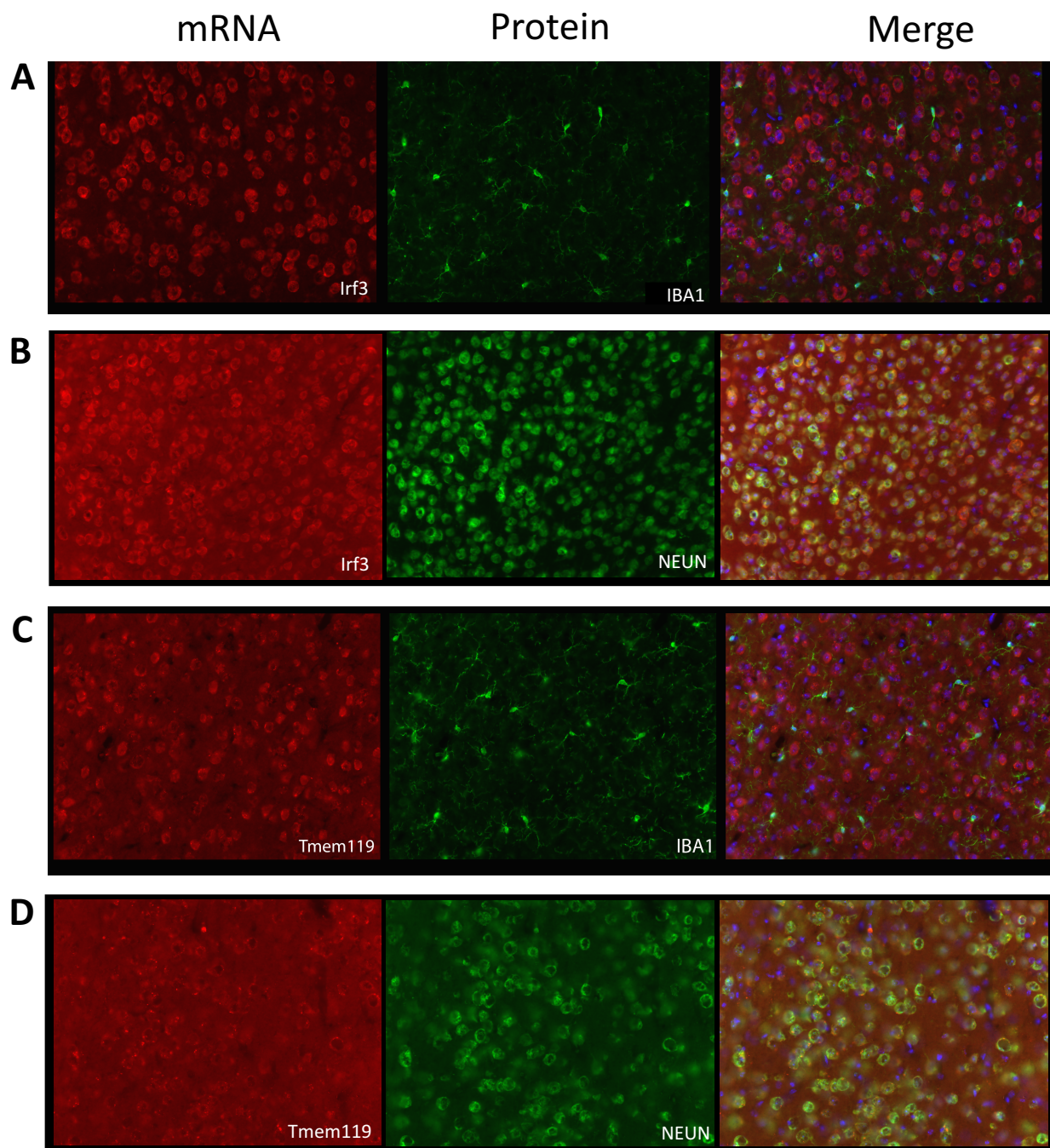

Supplement: Supplementary file 6 [file f1000research-6-13022-s0005.tgz › 8a6a17c8-d707-4559-9b8a-31cde6a1d003.pdf]
