# Supplementary material for: CNS cell-type localization and LPS response of TLR signaling pathways
Source: F1000Res. 2017 Jul 19;6:1144. [Version 1] doi: 10.12688/f1000research.12036.1 (PMC5621151; doi:10.12688/f1000research.12036.1)
Supplement: Supplementary file 7 [file f1000research-6-13022-s0006.tgz › 4db50d20-3d3c-44a6-aca8-8e7356b97016.pdf]

Table S1: Taqman Assays

Gene Name	Gene Expression Assay
18s	4333760T
Ccl5	Mm01302427_m1
Cd14	Mm00438094_g1
Cd68	Mm03047340_m1
Cxcl10	Mm00445235_m1
IFNb	Mm00439552_s1
Ikbkb (Ikkb)	Mm01222247_m1
Ikbke (Ikke)	Mm00444862_m1
Il1b	Mm00434228_m1
Il6	Mm00446190_m1
Irak1	Mm0119538_m1
Irak4	Mm00459443_m1
Irf3	Mm00516784_m1
Itgam (Cd11b)	Mm0434455_m1
Myd88	Mm00440338_m1
Rbfox3 (Neun)	Mm01248781_m1
Slc1a3 (Glast)	Mm00600697_m1
Tek	Mm00443243_m1
Ticam1 (Trif)	Mm00844508_s1
Tlr2	Mm00442346_m1
Tlr3	Mm01207404_m1
Tlr4	Mm00445273_m1
Tnf	Mm00443258_m1
Traf3	Mm00495752_m1
Traf6	Mm0493836_m1
