# Supplementary material for: CNS cell-type localization and LPS response of TLR signaling pathways
Source: F1000Res. 2017 Jul 19;6:1144. [Version 1] doi: 10.12688/f1000research.12036.1 (PMC5621151; doi:10.12688/f1000research.12036.1)
Supplement: Supplementary file 8 [file f1000research-6-13022-s0007.tgz › bbdf3528-a9aa-4c6c-9f59-563b14c06a73.pdf]

Table S2: Antibodies

Primary Antibodies for Westerns								
**Antibody**	**RRID**	**Manufacturer**	**Catalog #**	**Host**	**Clonality**	**Dilution**	**Diluant**	**predicted MW**
GFAP	AB_138737	Invitrogen	18-0063	Rabbit	polyclonal	1:5000	5% NFDM	50 kD
Iba1	AB_839504	Wako	019-19741	Rabbit	polyclonal	1:1000	5% NFDM	17 kD
IKKi (M-17)	AB_649212	Santa Cruz	sc-5693	Goat	polyclonal	1:200	5% NFDM	80 kD
IKKβ (P-20)	AB_2122157	Santa Cruz	sc-34673	Goat	monoclonal	1:200	5% NFDM	87 kD
IL-1β (H-153)	AB_2124476	Santa Cruz	sc-7884	Rabbit	polyclonal	1:200	5% NFDM	31 kD
IRAK-1 (F-4)	AB_2249106	Santa Cruz	sc-5288	Mouse	monoclonal	1:200	5% NFDM	80 kD
IRF-3 (FL-425)	AB_2264929	Santa Cruz	sc-9082	Rabbit	polyclonal	1:200	5% NFDM	50 kD
MYD88 (HFL-296)	AB_2146724	Santa Cruz	sc-11356	Rabbit	polyclonal	1:200	5% BSA	33 kD
TLR2 (H-175)	AB_2303458	Santa Cruz	sc-10739	Rabbit	polyclonal	1:200	5% NFDM	90-100 kD
TLR4 (25)	AB_10611320	Santa Cruz	sc-293072	Mouse	monoclonal	1:200	5% NFDM	95/120 kD
TRAF6 (H-274)	AB_793346	Santa Cruz	sc-7221	Rabbit	polyclonal	1:200	5% NFDM	60 kD
TLR3	AB_2287483	Enzo	ALX-804-362	Mouse	monoclonal	1:1000	5% NFDM	117 kD
TLR4	AB_839000	Novus	NB100-55951	Mouse	monoclonal	1:500	5% NFDM	95 kD
MyD88 (F-19)	AB_2146726	Santa Cruz	sc-8197	Goat	polyclonal	1:200	5% NFDM	33 kD
MyD88 (E-11)	AB_1126429	Santa Cruz	sc-74532	Mouse	monoclonal	1:200	5% NFDM	33 kD
MyD88	AB_302807	Abcam	Ab2064	Rabbit	polyclonal	1:1000	5% NFDM	33 kD
MyD88	AB_2146694	Abcam	Ab2068	Rabbit	polyclonal	1:1000	5% NFDM	33 kD
NeuN	AB_2532109	Abcam	ab177487	Rabbit	monoclonal	1:10,000	5% NFDM	34 kD
Traf6 (D-10)	AB_628391	Santa Cruz	sc-8409	Mouse	monoclonal	1:200	5% NFDM	60 kD
IRAK4	AB_10839262	Cell signaling	4363	Rabbit	polyclonal	1:1000	5% BSA	55 kD
IKKI (A-11)	AB_10989201	Santa Cruz	sc-376114	Mouse	monoclonal	1:200	5% NFDM	80 kD

Secondary Antibodies for Westerns						
**Antibody**	**RRID**	**Manufacturer**	**Catalog #**	**Host**	**Clonality**	**Dilution**
goat anti-rabbit IgG-HRP	AB_650500	Santa Cruz	sc-2301	goat	polyclonal	1:1000
goat anti-mouse IgG-HRP	AB_650499	Santa Cruz	sc-2302	goat	polyclonal	1:1000
donkey anti-goat IgG-HRP	AB_631728	Santa Cruz	sc-2020	donkey	polyclonal	1:1000
