# Supplementary material for: CNS cell-type localization and LPS response of TLR signaling pathways
Source: F1000Res. 2017 Jul 19;6:1144. [Version 1] doi: 10.12688/f1000research.12036.1 (PMC5621151; doi:10.12688/f1000research.12036.1)
Supplement: Supplementary file 9 [file f1000research-6-13022-s0008.tgz › 71d3fa52-fbca-4e23-b168-4bd4ca1c5feb.pdf]

Table S3: In situ probes and IHC antibodies

A

Primary Antibodies for Immunohistochemistry							
**Antibody**	RRID	**Manufacturer**	**Catalog #**	**Host**	**Clonality**	**Dilution**	**Diluant**
IRAK-1 (H-273)	AB_2233753	Santa Cruz	sc-7883	Rabbit	polyclonal	1:50	2% Goat Serum
MyD88 (F-19)	AB_2146726	Santa Cruz	sc-8197	Goat	polyclonal	1:250	2% Donkey Serum
TRAF6 (H-274)	AB_793346	Santa Cruz	sc-7221	Rabbit	polyclonal	1:50	2% Goat Serum
NEUN	AB_2298772	Millipore	MAB377	Mouse	monoclonal	1:500	2% Goat/Donkey Serum
**Secondary Antibodies for Immunohistochemistry**							
**Antibody**	RRID	**Manufacturer**	**Catalog #**			**Dilution**	
Alexa Fluor 568 donkey anti-goat	AB_142581	Thermo Scientific	A-11057	donkey	polyclonal	1:1000	
Alexa Fluor 488 donkey anti-mouse	AB_141607	Thermo Scientific	A-21202	donkey	polyclonal	1:1000	
Alexa Fluor 594 goat anti-mouse	AB_2534091	Thermo Scientific	A-11032	goat	polyclonal	1:1000	
Alexa Fluor 488 goat anti-rabbit	AB_2576217	Thermo Scientific	A-11034	goat	polyclonal	1:1000	

B

**mRNA**	**Custom Probe Sequence**	**Probe Label**	**Probe Concentration**	**Probe Hybridization Temperature**	**Primary antibody and dilution**	**Secondary antibody and dilution**
Irf3	AGA CTG AGC CTT GTA GAA TAA	5' and 3' DIG	100nM	54°C	Anti-DIG; 1:500 (Roche # 11 333 089 001)	Donkey anti sheep (594); 1:1000 (Life Technologies #)
Tmem119	ATA TCC ACA TCC GAA GAG CTG A	5' and 3' DIG	120nM	54°C	Anti-DIG; 1:500 (Roche # 11 333 089 001)	Donkey anti sheep (594); 1:1000 (Life Technologies #)

C

**Protein**	**Primary antibody and dilution**	**Secondary antibody and dilution**
Iba1	Anti-IBA1; 1:1000 (WAKO)	Donkey anti rabbit (488); 1:1000 (Life Technologies #)
NeuN	Anti-NeuN; 1:1000 (Millipore #MAB377)	Donkey anti mouse (488); 1:1000 (Life Technologies #)
