# Supplementary material for: CNS cell-type localization and LPS response of TLR signaling pathways
Source: F1000Res. 2017 Jul 19;6:1144. [Version 1] doi: 10.12688/f1000research.12036.1 (PMC5621151; doi:10.12688/f1000research.12036.1)
Supplement: Dataset containing the following six files: ISH localization images, ISH images, knockout animal qPCR, Sal-LPS qPCR data, Sal-LPS western blots and validation western blots [file f1000research-6-13022-s0000.tgz › c48cb01b-2cdf-4b48-88c3-397485e22218_Dataset_1.zip!/Dataset 1/ISH images.zip!/ISH images/Summary of fluorescent microscope images/IRF3 ISH testing summary 5.docx]

| Image | Brain region | U6 probe | U6 1**°** | U6 2**°** | Protein 1**°** | Protein 2**°** | Probe concen. | Slice size (uM) | Sudan Black |
| --- | --- | --- | --- | --- | --- | --- | --- | --- | --- |
| 1 | Cortex | + | + | + | IBA1 + | + | 2nM | 20 | + |
| 2 | Non-cortex | + | + | + | IBA1 + | + | 1nM | 20 | + |
| 3 | cortex | + | + | + | IBA1 + | + | 4nM | 30 | - |
| 4 | cortex | + | + | + | NeuN + | + | 1nM | 20 | + |
| 5 | Non-cortex | + | + | + | NeuN | + | 2nM | 20 | + |
| 6 | Non-cortex | + | + | + | NeuN + | + | 4nM | 30 | - |

**Merged**

**Protein**

**U6 snRNA**

1

2

3

4

5

6
