# Supplementary material for: CNS cell-type localization and LPS response of TLR signaling pathways
Source: F1000Res. 2017 Jul 19;6:1144. [Version 1] doi: 10.12688/f1000research.12036.1 (PMC5621151; doi:10.12688/f1000research.12036.1)
Supplement: Peer review history

# Peer review: Referee response for version 1

**DOI:** 10.5256/f1000research.13022.r26347

**Published:** 2017-09-27

**Authors:** Miles Herkenham

**Affiliations:** 1 National Institute of Mental Health, National Institutes of Health, Bethesda, MD, USA

I recommend adding text to the Discussion to include supporting data for the conclusion that TLR4, TLR2, and their related signaling components shown in Figure 1 are localized primarily to microglia, secondarily to vascular endothelia, and least in neurons. There are several sources of supporting data that should be described and cited. First, and the most expansive, is the RNA-Seq database available at the Barres Lab Stanford RNA-Seq Transcriptome website at https://web.stanford.edu/group/barres_lab/brain_rnaseq.html. The database is derived from the work published by Zhang *et al.*, 2014 (ref 25). The interactive site provides gene expression levels of most genes of interest, including the TLRs and all the signaling components shown in Figure 1. In agreement with the data in the McCarthy study, *Tlr4 *mRNA expression levels, provided in values of fragments per kilobase of exon per million reads mapped (FPKM), are highest in microglia (3), second in endothelia (2.3), and very low in all other cell types (~ 0.3-0.4 on astrocytes and OPCs, and ~ 0.1 on neurons). *Tlr2* mRNA is abundantly and almost exclusively expressed in microglia (>150). All of the downstream co-factors and pathways are predominantly expressed in microglia, with the exception of the side-chain pathway via TRAF3 and IRF3, which is expressed in all the cell types. The cytokine mRNAs measured by McCarthy, and others such as IL-1b and TNFα, are almost exclusively expressed in microglia. Finally, for TLR3, the FPKM values are astrocytes (13), endothelia (9), microglia (2.5), and neurons (< 1.0). 

 Next, the authors should note that earlier *in situ* hybridization studies showed *Tlr4 *mRNA expressed in a variety of blood-brain barrier cell types including choroid plexus epithelial cells, meningeal cells, vascular endothelial cells (Laflamme and Rivest, 2001, DOI: 10.1096/fj.00-0339com; Chakravarty and Herkenham, 2005, DOI: 10.1523/JNEUROSCI.4268-04.2005), and within the brain, microglial cells (Chakravarty, 2005).  Supporting the microglial localization, a recent article used the very sensitive and specific RNAscope (ACD) colorimetric methodology to co-localize *Tlr4* mRNA and *Iba1* mRNA (Kashima, 2017, DOI: 10.1073/pnas.1705974114) and showed that in the nucleus accumbens, the microglia-TLR4 double-labeled population was the majority (~80%) of all Tlr4 mRNA-positive cells. Finally, the early *in situ* hybridization autoradiographic work also showed that the TLR4 mRNA expression level was downregulated by LPS at 3 h post-injection (Laflamme and Rivest, 2001), in support of McCarthy’s findings with qPCR.

 A TLR4-bearing cell population overlooked by McCarthy *et al.* in terms of LPS responsiveness is endothelial cells, which strongly express *Tlr4* mRNA. These cells are the major first responders to LPS (Serrats, 2010, DOI: 10.1016/j.neuron.2009.11.032). It is not clear whether this population survived the separation procedure used by McCarthy. 

 The LPS stimulation data in the report are supported by other published data, but the experiment was not performed optimally because the 24-h survival time selected was too long. There is an early response to LPS (0.5–2 h) that is chiefly mediated by cells at the blood-brain barrier, and later responses, indicated by induction of IkB and cytokine mRNAs within microglia (Quan, 1997, PMID: 9380746; Quan, 1998 , PMID: 10378870) might be prostaglandin- or cytokine-mediated (Serrats, 2010). Note that LPS does not significantly cross the blood brain barrier. At 12 h post-LPS, most responses have died down, and at 24 h, the initial LPS-mediated effects have dropped to near zero (Quan, 1998; Serrats, 2010). 

 Nevertheless, the inclusion of LPS data raises another important observation that should be addressed—neurons are unresponsive to LPS. We reported that primary cultured mouse cortical and hippocampal neurons do not show activation of NF-kB pathways by LPS, indicating that they do not possess functional TLR4 (Listwak, 2013, DOI: 10.1016/j.neuroscience.2013.07.013). Microglia, in contrast, have a massive response to LPS.

 McCarthy *et al.* importantly address the lack of specificity of antibodies. That conclusion is also supported by our work on the NF-kB pathways. We attacked that problem in our study showing that many of the published antibodies for the NF-kB subunits p65 and p50 and their activated (phosphorylated) forms were nonspecific (Herkenham, 2011, DOI: 10.1186/1742-2094-8-141). The use of these antibodies had supported the claim in the literature that NF-kB is active in neurons. Thus, we heartily agree that antibodies are not specific in many studies of TLR pathways, especially for immunohistochemistry and especially when epitope levels are low. We endorse the increased demand for rigor in use of antibodies imposed by many journals and by the NIH grant review process. It would be helpful if McCarthy could mention these new demands for rigor, which will raise awareness of the burdens caused by improper antibody use.

 My main objection to McCarthy study is the poor quality of their *in situ *hybridization based on use of locked nucleic acid (LNA) probes and digoxygenin detection. I would recommend that future work be done with the new highly sensitive and selective probes from ViewRNA (Affymetrix) or RNAscope (ACD).

I have read this submission. I believe that I have an appropriate level of expertise to confirm that it is of an acceptable scientific standard.

# Peer review: Referee response for version 1

**DOI:** 10.5256/f1000research.13022.r24588

**Published:** 2017-08-16

**Authors:** Okun Eitan

**Affiliations:** 1 The Mina and Everard Goodman Faculty of Life Sciences, Bar-Ilan University, Ramat Gan, Israel

In this paper, Gizelle, Courtney, Yuri and Adron have undertook a difficult task of determining the expression pattern of Toll-like receptors (TLRs), their adapter proteins and downstream effector proteins in CNS cells. This is an important and timely work which emphasizes the importance of establishing the appropriate tools in order to advance an entire field.

 After reading the manuscript, I'd like to raise the following points:

1. Technical comment: under "tissue harvest and microglial isolation", it is described that the used kit for tissue dissociation uses papain. Is it possible that this type of dissociation degraded some of the extracellular epitopes and altered their expression in subsequent western blots? Perhaps it should be cautioned that different enzymes or dissociation methods can result in different outcomes.
2. Under "protein isolation and western blots", it is mentioned that appropriate loading controls could not be found for all cell types. This is understandable, as specialized CNS cells contain different levels of proteins such as beta-actin, alpha tubulin, beta tubulin and others. Please indicate which proteins were tested in your hands.
3. With respect to the probes used for *in-situ* hybridization, it is entirely possible that different cells will express different splice variants of the genes. Has that been taken into consideration?
4. On page 6, results section, it is unclear to me why there is a lack of NeuN expression in the CD11-/ACSA2- fraction (Figure 2C). Should it not contain neuronal cells?
5. On the 2nd results paragraph, the fold change in expression of the different genes is indicated. I think that it should be further stressed that despite the fact that fold changes are important to note, the very expression, even though it is in smaller levels, of TLRs 2,3 and 4 is present in non-microglial cells.
6. On page 7, under "antibody validation in knockout tissue and HEK-293 cells". It is debatable whether post-translational modification can alter the size of a protein in a gel. Also, a KO tissue which lacks the target of the antibody can still express a target which will be second-in-highest affinity in absence of the original target. therefore, overexpressing the target of interest (TLR2,3,4 etc fused to GFP) will determine clearly whether the commercial antibody is capable of binding its target. This way, if the antibody cannot bind its target TLR but GFP can be detected, this is a definite proof that the antibody fails to detect its target under these conditions.
7. Table S2 should also include specification of the declared application for the antibody by the manufacturer, whether it is denaturative WB, IHC, ELISA etc...
8. On the same note, although this information is often missing from datasheets of commercial antibodies, the epitope used to vaccinate against should be indicated.
9. On page 13, the issue of a microglial-specific marker, *Tmem119*, not being detected on microglail cells using the described protocol is of concern. I understand that 0.1% Tween-20 was used. Is it possible that a more harsh detergent at a higher concentration would solve this issue? For example - 0.5-1% triton-x?
10. In the discussion, the issue of expression of adapter-protein expression in various cells is discussed. Despite the fact that MyD88 is a signaling mediator of the IL1R family, which is also reported to be abundantly expressed in the brain, it is not discussed in the paper. Therefore, the expression of certain signaling molecules could be the result of pathways unrelated to TLRs but rather to other immune receptors such as IL1R.
11. On the last sentence on page 15, the authors rightfully, and correctly indicated that the current analysis cannot take into consideration any brain-region specific expressions, and that the probes used cannot address alterations in splice variants. This by itself could draw the conclusion that a similar effort should concentrate at a specific TLR at a time, studying the different splice variants in details throughout the brain.

Minor points:

1. Page 4, left column, the end of the second paragraph - a reference has to be added following "expression in neurons".
2. Same page, right column, lines 3-4, immune activation is exemplified by LPS. I think that it should be stated that it is TLR4-specific immune activation, as there are numerous ways to activate the immune response by different pathways.
3. Please abbreviate "MS" in page 5, left column: "eluted using MS columns".
4. Same page, right column, please abbreviate "NBF": "in 10% NBF overnight.."
5. On page 16, first sentence, i think that it is better to replace 1:1 with 'correlative', as 1:1 relates to quantifiable ratios, whereas the agreement between mRNA to proteins is describes as trends.

I have read this submission. I believe that I have an appropriate level of expertise to confirm that it is of an acceptable scientific standard.
